# Recruitment, regulation, and release: Control of signaling enzyme localization and function by reversible S-acylation

**DOI:** 10.1016/j.jbc.2024.107696

**Published:** 2024-08-19

**Authors:** Xiaotian Zhang, Gareth M. Thomas

**Affiliations:** Department of Neural Sciences, Center for Neural Development and Repair, Philadelphia, Pennsylvania, USA

**Keywords:** palmitoylation, DLK, JNK, retrograde signaling, protein trafficking, axon degeneration, neurodegeneration, axon transport, synapse, cancer, oncogene, immunity, immune signaling, inflammation

## Abstract

An ever-growing number of studies highlight the importance of S-acylation, a reversible protein-lipid modification, for diverse aspects of intracellular signaling. In this review, we summarize the current understanding of how S-acylation regulates perhaps the best-known class of signaling enzymes, protein kinases. We describe how S-acylation acts as a membrane targeting signal that localizes certain kinases to specific membranes, and how such membrane localization in turn facilitates the assembly of signaling hubs consisting of an S-acylated kinase’s upstream activators and/or downstream targets. We further discuss recent findings that S-acylation can control additional aspects of the function of certain kinases, including their interactions and, surprisingly, their activity, and how such regulation might be exploited for potential therapeutic gain. We go on to describe the roles and regulation of de-S-acylases and how extracellular signals drive dynamic (de)S-acylation of certain kinases. We discuss how S-acylation has the potential to lead to “emergent properties” that alter the temporal profile and/or salience of intracellular signaling events. We close by giving examples of other S-acylation–dependent classes of signaling enzymes and by discussing how recent biological and technological advances should facilitate future studies into the functional roles of S-acylation–dependent signaling.

## Overview: Why is S-acylation a unique form of protein-lipid modification?

Eukaryotic cells use three major mechanisms to lipidate proteins, N-myristoylation, prenylation, and S-acylation. N-myristoylation usually involves the addition of C14 myristate to the N-terminal glycine residue of proteins, following cleavage of the initiating methionine (1, 2). More rarely, a newly exposed N-terminal glycine can be myristoylated following caspase cleavage of the parent protein (3). Two related enzymes, N-myristoyltransferase-1 and N-myristoyltransferase-2 (NMT1, NMT2), catalyze N-myristoylation in mammals. Protein prenylation involves lipidation of a C-terminal cysteine within a CaaX motif, followed by cleavage of the aaX tripeptide and subsequent methylation of the newly exposed prenylcysteine. Prenylation can be further subdivided into farnesylation and geranylgeranylation, catalyzed by farnesyltransferase, and by geranylgeranyltransferase-1 and geranylgeranyltransferase-2, respectively (4).

**Figure 1:**
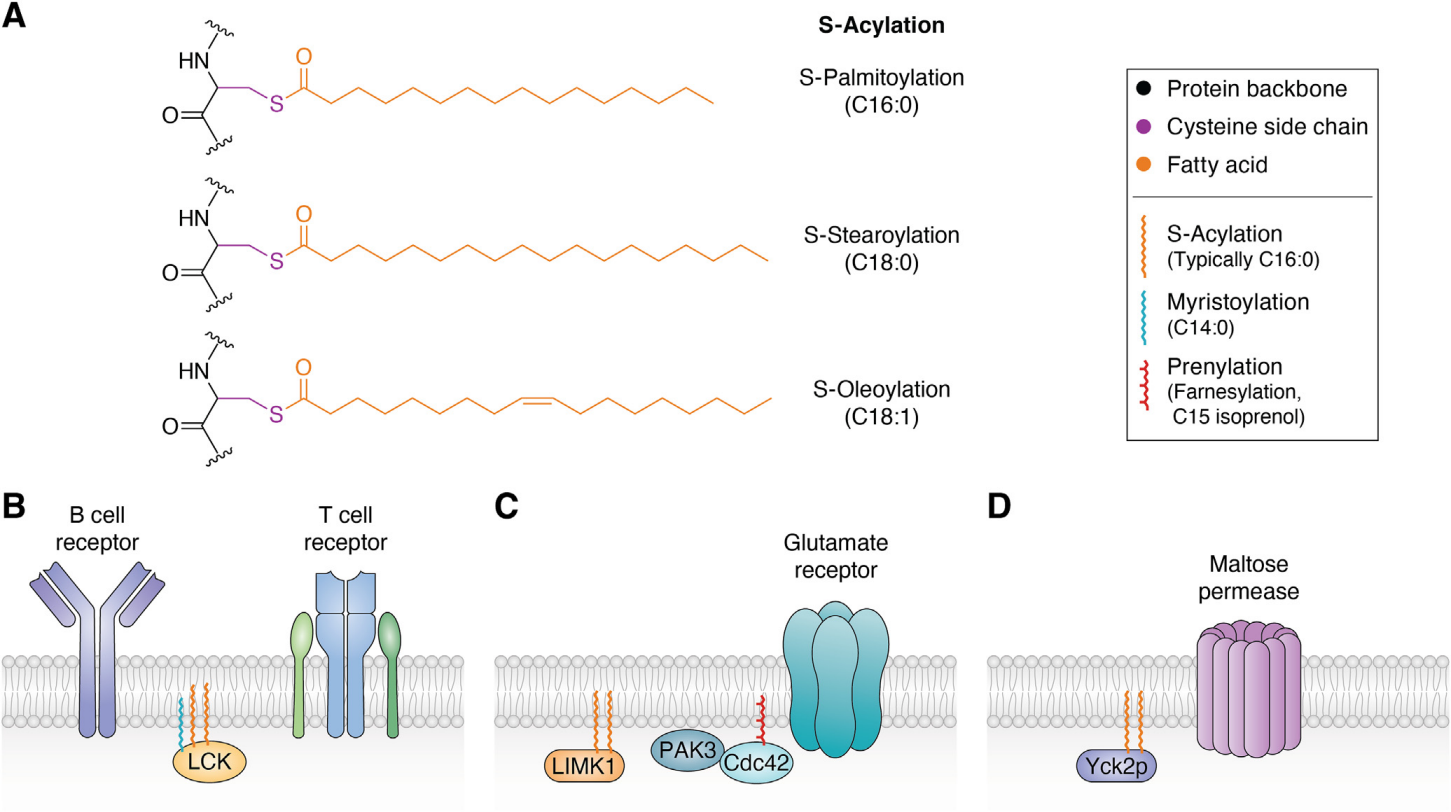
**S-acylation localizes protein kinases close to upstream activators and/or downstream targets.***A*, examples of different types of S-acylation. Protein cysteine residues (*purple*) are most commonly modified by C16:0 (palmitate) but can also be modified by C18:0 (stearate), C18:1 (oleate), and potentially other long-chain fatty acids (*orange*). *B–D*, roles of S-acylation in assembly and organization of membrane-associated signaling complexes. *B*, S-acylation localizes Lck close to T cell and B cell receptors (TCR and BCR). *C*, S-acylation targets LIM kinase-1 (LIMK1) close to its upstream activator p21-activated protein kinase-3 (PAK3) and PAK3’s regulator, the lipidated small G protein Cdc42. *D*, S-acylation localizes the yeast casein kinase Yck2p close to the maltose permease. The *lower panel* of the box shown in *A* diagrams structures of different types of protein-lipid attachment. A second type of prenylation, geranylgeranylation, is not shown.

Myristoylation and prenylation are critically important for cellular function but are irreversible and occur within defined consensus sequences. These two features contrast with S-acylation, which involves modification of protein cysteine residues with a long-chain fatty acid *via* a thioester bond (5). S-acylation is often referred to as protein (or *S*-) palmitoylation because the fatty acid is typically palmitate (C16:0, fully saturated). However, other lipids (*e.g.*, C18:0; stearate, C18:1; oleate) can also be similarly attached (6, 7) (Fig. 1*A*), so the term S-acylation is more accurate and will be used in this review. Importantly, S-acylation differs from myristoylation and prenylation because the lability of the thioester bond makes this modification reversible; S-acyl chains are added to target proteins by a family of zinc finger Asp-His-His-Cys motif (ZDHHC)-domain–containing protein acyltransferases (PATs, 23 in humans) (8, 9, 10), and removed by alpha-beta hydrolase domain (ABHD)-containing de-S-acylases (also known as depalmitoylases or thioesterases) (9, 10).

In addition to its potential reversibility, other aspects of S-acylation–dependent regulation allow additional levels of control, compared to other protein-lipid modifications. For example, NMT1, NMT2, and farnesyl and geranylgeranyltransferases are ubiquitously expressed, whereas expression of certain PATs and ABHD de-S-acylases is restricted to specific cell types (11). A given protein may thus only become S-acylated, or its S-acyl chain may only be removed, if a cell expresses the cognate PAT or de-S-acylase, respectively. In addition, even within a single cell type, different PATs reside in different subcellular locations (12, 13, 14), so the initial S-acylation event and, thus, the likely initial membrane to which the S-acylated substrate is targeted, may also differ depending on the PAT(s) expressed in a given cell. The same is true for ABHD de-S-acylases. Moreover, catalytic activity, subcellular localization, and/or protein–protein interactions of both PATs and de-S-acylases can potentially be regulated by extracellular signals or by other changes in the environment, providing additional layers of control of S-acylation of specific substrate proteins (15).

Another factor that sets S-acylation apart from other forms of protein lipidation is that while myristoylation and prenylation occur at defined N- and C-terminal consensus sequences, respectively, S-acylation can potentially occur at any surface-exposed cysteine. Although no precise consensus sequence has been identified for S-acylation, many S-acylated sites have common features—they are often found close to other sites of lipidation (myristoylation, prenylation) and, when those modifications are absent, are often located close to the cytosolic boundary of (or just within) transmembrane segments, and/or in regions containing basic and/or hydrophobic residues (16).

The greater variety of potential S-acylation sites, compared with myristoylation and prenylation, confers additional regulatory potential. For otherwise soluble proteins, perhaps the simplest effect of S-acylation is to increase protein hydrophobicity and thereby enhance membrane interactions. When a protein becomes lipidated close to its N or C terminus, this region may then become membrane-associated, while other domains of the protein “dangle” into the cytosol. Myristoylation, prenylation, and S-acylation all have the potential to control protein localization in this way. However, S-acylation can also occur far from the N or C termini of a protein, potentially affecting not only the membrane targeting but also other properties of that protein. For example, the S-acylated form may undergo different protein–protein interactions and, if it is an enzyme, may have altered activity.

It is perhaps no surprise then, that increasing numbers of reports highlight the importance of S-acylation not only for signaling enzyme localization but also for activity and function. Here, we highlight recent progress in this field, focusing on how S-acylation affects perhaps the best-known subclass of signaling enzymes, protein kinases. We then briefly summarize roles for S-acylation in the regulation of other signaling enzymes and pathways.

### S-acylation targets protein kinases to membranes

The first reports of S-acylated protein kinases emerged from studies of the Src family kinases (SFKs) (17, 18, 19). Several SFKs, though not Src itself, are S-acylated close to their myristoylated N-terminal glycine (after cleavage of the initiating methionine). The best-studied S-acylated SFKs are Fyn and Lck, whose S-acylation is necessary for their stable membrane attachment. S-acylation is also necessary for B and T cell receptors (BCR, TCR) signaling mediated by both Fyn and Lck (20, 21, 22), suggesting that a key role of S-acylation is to localize these SFKs close to BCRs, TCRs, and potentially other transmembrane receptors (Fig. 1*B*).

S-acylation also controls membrane localization of LIM kinase-1 (LIMK1), a kinase heavily implicated in the control of intracellular actin dynamics *via* its phosphorylation of the cofilin family of proteins (23, 24, 25). LIMK1 lacks any additional myristoylation consensus and so is targeted to membranes by S-acylation at C5, C6 alone (23). In neurons, S-acylation targets LIMK1 to the membrane of dendritic spines—specialized, actin-enriched structures that are the postsynaptic site of many excitatory synapses and whose regulation is linked to higher brain functions such as learning and memory (23, 26, 27). Consistent with its spine-enriched targeting, the S-acylated form of LIMK1 is essential for changes in dendritic spine size in response to elevated neuronal activity (23). Moreover, an intriguing recent study using a form of LIMK1 that becomes activated “on demand” in the presence of a small molecule, suggested that LIMK1 activity is sufficient to drive increases in synaptic transmission and improvements in spatial memory (28). Though not directly tested, it is tempting to speculate that these recently described roles of LIMK1 involve its S-acylated, spine-targeted form, which is perfectly positioned to govern changes in synaptic efficacy.

Another dually lipidated kinase is Ca(2+)/calmodulin-dependent protein kinase–like CREB kinase-III (CL3), also known as CAMKIγ (29). CL3 activity controls the growth of neuronal dendrites and axons, an activity that requires initial prenylation and then subsequent S-acylation. The former modification targets CL3 to the Golgi, while the latter is critical for CL3 to associate with lipid raft microdomains to assemble a “signalosome” that drives CL3-dependent neuritogenesis (30).

### Targeting of kinases to membranes is an evolutionarily conserved function of S-acylation

S-acylated kinases are also not limited to mammalian cells. For example, several fundamental discoveries related to S-acylation were first made in yeast, including the initial characterization of PATs (31, 32, 33) and the finding that one of these enzymes, Akr1p, S-acylates the casein kinase Yck2p (34), thereby targeting Yck2p to the plasma membrane. Regulation of casein kinases by S-acylation is highly evolutionarily conserved; mammalian casein kinase isoforms such as casein kinase I-gamma (CK1γ) are S-acylated at a similar C-terminal region, which again controls membrane targeting (35, 36). As discussed in more detail later, S-acylated protein kinases are also found in plants. These include the rice (*Oryza sativa*) calcium-dependent protein kinase, which requires dual myristoylation and S-acylation for stable association with the plasma membrane (37). There are thus several examples across all branches of eukaryotes that S-acylation is critical to target a key subset of otherwise soluble kinases to membranes.

### S-acylation targets kinases to membrane-localized signaling hubs

It is perhaps not surprising that S-acylation–dependent membrane targeting of specific kinases localizes those kinases close to their upstream activators and/or downstream targets. One well-known example is the transmembrane TCR and BCR complexes that drive Lck/Fyn activation (20, 21, 22) (Fig. 1*B*). Transmembrane channels and receptors such as glutamate receptors and TrkB likewise play central roles in the regulation of S-acylated LIMK1 and CL3, respectively (23, 29). S-acylation can also position kinases close to other lipid-modified and/or membrane-associated cytosolic signaling proteins. For example, activation of LIMK1 involves the p21-activated protein kinase-3 (PAK3), which is itself recruited to the dendritic spine membrane by lipidated Cdc42/Rac/Rho family small G proteins (23, 38) (Fig. 1*C*). Rho family small G proteins are also involved in CL3 signaling, but this time as downstream targets activated by the guanyl nucleotide exchange factor Sif and Tiam1-like exchange factor (STEF, also known as T cell lymphoma invasion and metastasis-2), whose membrane localization is influenced by binding to phosphoinositide lipids (30). In yeast, S-acylated Yck2p regulates the transmembrane maltose permease Mal61 (39) (Fig. 1*D*). The conclusion that S-acylation of protein kinases facilitates assembly and activation of membrane-associated signaling hubs, thus also appears to hold across a variety of organisms.

### S-acylation facilitates long-distance kinase signaling by vesicular hitchhiking

In the above examples, S-acylation–dependent membrane targeting ensures spatially precise, highly localized protein kinase signaling. However, intracellular signals must sometimes be transferred over long distances, and it is now clear that S-acylation is also critical for this very different form of signaling. In particular, neurons in the peripheral nervous system extend their axons up to 1 m in humans to connect with their final targets. Because only a subset of axonal proteins are likely translated locally in the axon (40), many proteins that function in distal axons must hence be made in the cell body and trafficked all the way from the neuronal soma (41). Long-distance trafficking is even more of an issue for a subclass of axonal proteins termed retrograde signaling proteins. These proteins are also predominantly made in neuronal cell bodies and transported anterogradely to distal axons but must then retrace their steps to convey signals back from axons to the neuronal soma (42, 43). This bidirectional transfer of proteins between neuronal cell bodies and distal axons is essential both for correct neurodevelopment and for appropriate responses to axonal injury or damage (42, 44). It has recently become clear that S-acylation facilitates the long-distance transfer of retrograde signals, by allowing kinases to hitchhike on the surface of axonal vesicles (45, 46, 47, 48, 49). These S-acylated, vesicle-associated kinases can thereby take advantage of fast axonal transport to convey a response to a specific signal along axons far faster than would be achieved by the slow axonal transport used by cytosolic proteins, or by passive diffusion alone (50).

**Figure 2:**
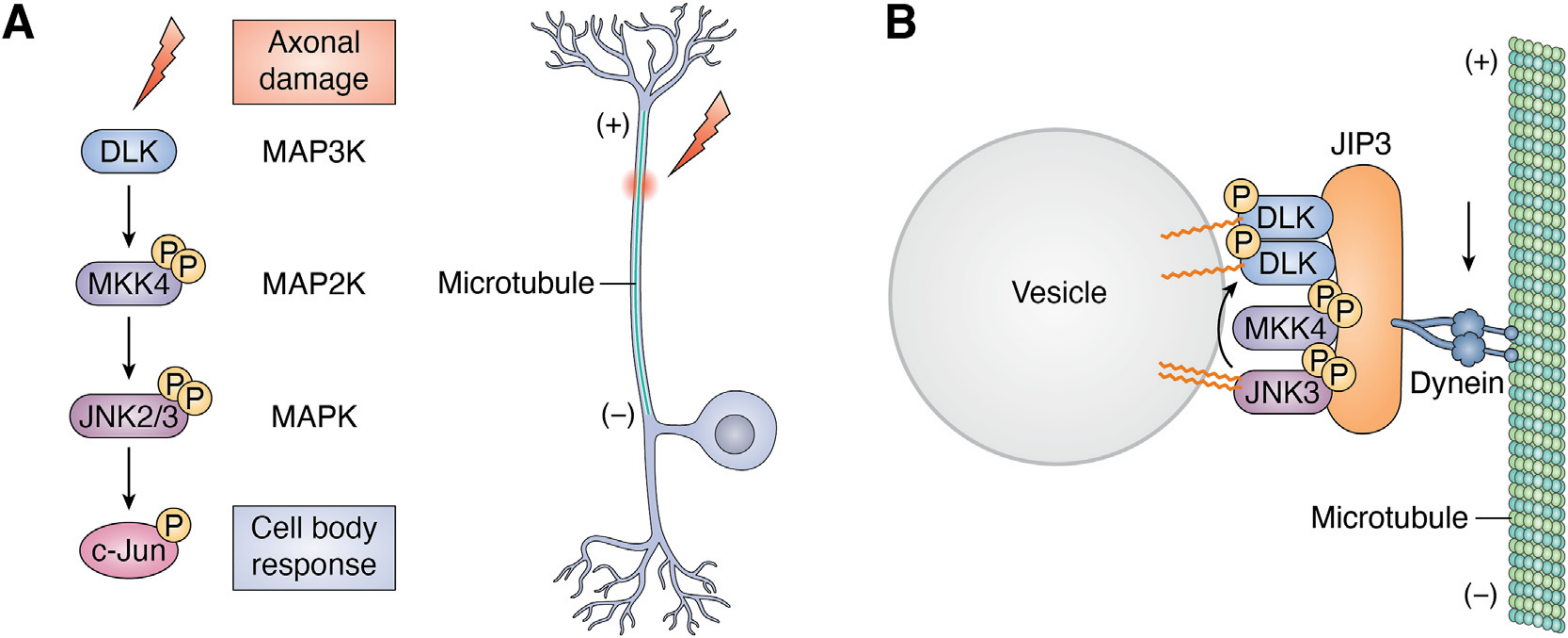
**S-acylation is critical for multiple properties of the DLK/JNK signalosome.***A*, after neuronal injury or other forms of axonal damage or stress (*red lightning bolt*), a MAP kinase module consisting of the MAP3K DLK, the MAP2K MKK4, and the MAPKs JNK2 and JNK3, conveys retrograde signals to the neuronal soma, resulting in phosphorylation of c-Jun and other transcription factors. The *right hand schematic* shows a pseudounipolar dorsal root ganglion (DRG) sensory neuron, a cell type in which DLK-JNK signaling has been extensively studied *e.g.*, (46, 49, 52, 147), but DLK-JNK signaling also plays key roles in more widely found multipolar neurons (56, 59, 60, 148). *B*, S-acylation targets DLK and JNK3, and likely MKK4 and the retrograde scaffolding protein JIP3, to the same population of axonal trafficking vesicles. JIP3 in turn binds the retrograde motor protein dynein that controls microtubule-dependent transport. S-acylation of DLK is also essential to establish a positive feedback loop, whereby JNK3 phosphorylates and further activates DLK, locking the module in a highly active state, which may be important for the ability of the DLK–MKK4–JNK3 pathway to convey retrograde signals over long times and distances. DLK, dual leucine-zipper kinase; JIP3, JNK-interacting protein 3; JNK, c-Jun N-terminal kinase; MAP3K, mitogen-activated protein kinase kinase kinase; MAPK, mitogen-activated protein kinase.

One key example of the functional importance of this axonal hitchhiking is during neurodevelopment, when sensory and sympathetic neurons compete for limited supplies of neurotrophins such as nerve growth factor (44, 51). Neurons that lose this competition for nerve growth factor do not die passively, but instead actively degenerate *via* a process that requires the mitogen-activated protein kinase kinase kinase dual leucine-zipper kinase (DLK) (52, 53, 54). Trophic factor deprivation of axons of cultured dorsal root ganglion (DRG) sensory neurons triggers DLK-dependent phosphorylation of the downstream mitogen-activated protein c-Jun N-terminal kinases (JNK, MAPK), which then phosphorylates transcription factors including c-Jun and activating transcription factor-2 in neuronal cell bodies (52) (Fig. 2*A*). It was previously unclear how DLK, a predicted soluble diffusible kinase, could mediate this directional, long-distance signaling, but Holland and co-workers mapped a conserved S-acylation site in DLK (46). In a series of studies, they reported that S-acylation was critical for DLK targeting to axonal vesicles and for axon-to-soma signaling not only during neurodevelopment but also in the adult nervous system following axonal injury (46, 54). They further identified the PAT ZDHHC17 as critical for S-acylation and membrane localization of DLK in mammals, while two ZDHHC17 orthologs control membrane localization of the *Caenorhabditis elegans* DLK ortholog DLK-1 (54). Consistent with its assignation as a major PAT for DLK, ZDHHC17 loss impairs DLK-dependent retrograde signaling both in cultured neurons and *in vivo*, mimicking the effects observed with the loss of DLK S-acylation (54).

### An S-acylation–dependent vesicular signalosome

Just as S-acylation helps assembling signaling hubs at the plasma membrane, it can do the same for axonal vesicles. In mammals, DLK’s main “downstream” targets are the JNK family of MAPKs, particularly JNK2 and JNK3 (47, 52, 55). Indeed, several phenotypes observed in the absence of JNK2 and/or JNK3 strikingly resemble those seen following DLK loss, including protection against excitotoxin- and optic nerve injury–induced neuronal death (47, 56, 57, 58, 59, 60). Recent reports potentially explain this specific coupling: JNK2 and JNK3 undergo S-acylation at their C-terminal extensions (47, 61, 62), while JNK1 predominantly lacks this C-terminal extension and is not detectably S-acylated, at least in DRG sensory neurons, a cell type widely used to for studies of DLK-JNK signaling (47) (Fig. 2*A*). Similar to DLK, S-acylation directs JNK3 to axonal vesicles, many of which colocalize and cotraffic with DLK (47) (Fig. 2*B*). Additionally, ZDHHC17 is also likely the PAT that S-acylates JNK3 (54, 63), and DLK and JNK3 both contain sequences that broadly conform to ZDHHC-ankyrin binding motifs (zDABMs; (64, 65)), which are recognized by ZDHHC17’s ankyrin repeat region (64, 66). These findings suggest that specific features of the modifying PAT are important for DLK/JNK3 S-acylation and subsequent vesicle targeting. Consistent with this notion, mutation of its zDABM impacts DLK S-acylation and membrane association (54). Although, to our knowledge, the functional importance of JNK3’s zDABM has not yet been investigated, JNK3 can directly bind ZDHHC17 (67), and peptides derived from ZDHHC17’s ankyrin repeat region effectively prevent JNK3-ZDHHC17 binding and JNK3-dependent signaling (63). These findings suggest that specific interactions between ZDHHC17 and both DLK and JNK3 are important for their regulation by this PAT. Moreover, in neurons, both DLK and JNK3 also cotraffic with JNK-interacting protein 3 (JIP3), a scaffold for multiple JNK pathway kinases that is also required for DLK-dependent retrograde signaling (46, 47, 52) (Fig. 2*B*). Significantly, only wild type (S-acylation–competent), and not palmitoyl-mutant, DLK can bind to JIP3 (46). Together these findings suggest that S-acylation, initially mediated by ZDHHC17, facilitates assembly and cotrafficking of a DLK–JNK3–JIP3 signaling complex on axonal vesicles.

### S-acylation–dependent regulation of protein kinase activity

To this point, the critical roles for S-acylation in kinase-dependent signaling that we have summarized can largely be explained by this modification controlling kinase localization. However, as mentioned above, S-acylation is not restricted to protein N or C termini and thus has greater potential to influence other aspects of kinase function, including catalytic activity. While this is not the case for certain kinases, such as CK1γ3, which retains full activity in the absence of S-acylation (35), S-acylation, and activity of other kinases is far more intimately coupled. For example, G protein–coupled receptor (GPCR) kinase 6 displays greater activity, even *in vitro*, when S-acylated (68). This coupling of S-acylation and activity is even more striking for DLK, for which S-acylation not only acts as a vesicle targeting signal but also, unexpectedly, is essential for DLK to phosphorylate and activate its direct substrate MKK4 and, in turn, for DLK-dependent activation of the downstream MAPK JNK3 (46). This requirement is not due to subcellular localization because it can be replicated with purified DLK isolated from cells and subsequently assayed against purified MKK4 *in vitro* (46). One possible explanation for this unexpected finding is that S-acylation triggers a conformational change in DLK that facilitates its further activation, most likely by phosphorylation. However, in the absence of structural information and/or phospho-specific antibodies to monitor DLK’s activation state, insights into how S-acylation controls DLK activity remain limited.

**Figure 3:**
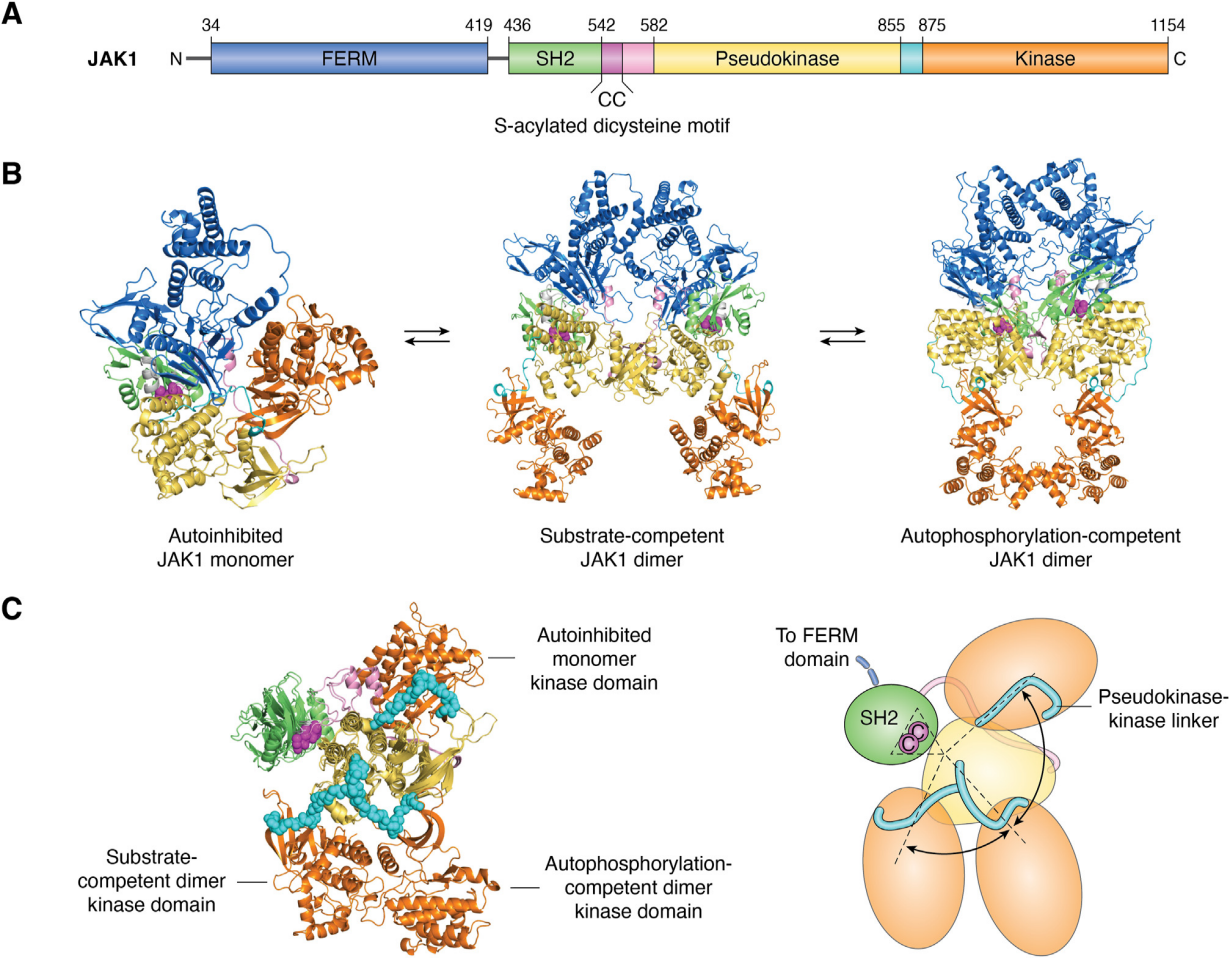
**S-acylation may control structural transitions that drive JAK1 activation.***A*, JAK1 domain structure. FERM: 4.1 protein, ezrin, radixin, moesin domain; CC: S-acylated dicysteine motif. *B*, JAK1 structures, color-coded based on *A*, suggest a potential equilibrium between autoinhibited monomeric human JAK1 model (*left*), substrate-competent JAK1 homodimer (*center*), and autophosphorylation-competent JAK1 dimer (*right*). *C*, when JAK1 C-terminal regions (SH2->kinase domain) are superimposed, the JAK1 S-acylated dicysteines lie in a region that resembles a fulcrum as JAK1 transitions between different forms (*left*: *ribbon model*; *right*: *simplified cartoon*), raising the possibility that S-acylation may favor certain transitions and disfavor others. This model is consistent with findings that acylation facilitates JAK1’s ability to transphosphorylate and to phosphorylate STAT3. JAK1, Janus kinase-1.

S-acylation–dependent control of DLK activity might thus be considered an anomalous finding, but more recently, a second kinase, Janus kinase-1 (JAK1; Fig. 3*A*), was also found to display S-acylation–dependent enzymatic activity not only in cells but also in *in vitro* assays (69). In this case, available phospho-specific antibodies allowed the authors to demonstrate that S-acylation is critical for transphosphorylation of key sites in JAK1’s activation loop. The authors used recent JAK1 structures and also generated alpha-fold models to reveal that JAK1 may exist in at least three states: an autoinhibited monomer, a dimer in which the kinase domains of the two JAK1 subunits face one another, and another dimer in which the kinase domains face outward (69) (Fig. 3*B*). These latter two structures may resemble forms of JAK1 that are more capable of autophosphorylation, or of exogenous substrate phosphorylation, respectively. Intriguingly, JAK1’s S-acylation sites lie in a region that appears to act as a fulcrum around which JAK1 domains rotate as JAK1 transitions between these different structural states (69) (Fig. 3*C*). Though not directly demonstrated, it is thus tempting to speculate that S-acylation permits or drives transitions toward the state(s) that favor JAK1 activation. However, it is important to note that an alternative (though not mutually exclusive) explanation is that S-acylation favors JAK1 transphosphorylation by changing a three-dimensional situation (free-floating JAK1 molecules that must find one another in the cytosol) to a two-dimensional one (membrane-tethered JAK1 monomers that are more likely to encounter one another). Importantly, though, LIMK1 behaves differently to DLK and JAK1, in that S-acylation is important for its signaling ability in neurons but is dispensable for its direct phosphorylation of substrates in *in vitro* assays (23). This mutual control of membrane association and kinase activity by S-acylation thus appears to be the exception rather than the rule, but it will nonetheless be of great interest to determine whether other kinases or other enzymes that undergo S-acylation close to their catalytic domains are subject to similar coordinated regulation.

Figure adapted from (69), with permission.

### Roles of dynamic S-acylation in stimulus-dependent kinase signaling

Despite demonstrating critical functional roles for S-acylation in a variety of contexts, none of the mechanisms described to this point take advantage of the unique reversibility of S-acylation. This attribute allows S-acylation to potentially occur “on demand,” for example, in response to a given extracellular or intracellular cue, while de-S-acylation has the potential to subsequently and rapidly revert a signaling system to its original state. Perhaps surprisingly, few examples of such regulation have been described for S-acylated kinases, but one exception is work from the Boehning and Akimzhanov labs, assessing signaling by the TCR and the proapoptotic Fas receptor. In a series of elegant studies, they found that stimulation of T cell–like cell lines with Fas ligand or TCR engagement results in rapid but transient (peaking at 2–5 min poststimulation) S-acylation of Lck (70, 71). Similar and even more rapid kinetics of S-acylation (peaking at 2 min post-TCR engagement) were demonstrated for the “upstream” tyrosine kinase ZAP-70, whose recruitment to TCR complexes and subsequent phosphorylation by Lck is essential for TCR-dependent signaling (72).

Recent reports have identified additional dynamically S-acylated kinases. For example, the MAPKs extracellular signal–regulated kinase 1 and 2 (ERK1 and ERK2) are S-acylated and their S-acylation is increased by EGF treatment and decreased by a metabolic stressor, a high concentration of palmitate (73). Pharmacological treatments that impact ERK S-acylation also affect ERK-dependent transcriptional responses (73), although whether the latter effect was directly due to alterations in ERK S-acylation (rather than other targets of these compounds) remains to be determined. Given the numerous roles of ERK in different cell types and contexts, additional insights into the regulation and functional consequences of ERK S-acylation are eagerly awaited.

Some of the most striking reports of dynamic S-acylation of kinases have come from studies on plants. In particular, exposure of Arabidopsis to flg22, a peptide derived from bacterial flagellin that mimics a microbial stimulus, results in rapid S-acylation of the plant receptor kinase (RK), flagellin sensing-2 (FLS2) (74). The authors further found that S-acylation is essential for FLS2-mediated immune signaling and resistance to a pathogenic bacterium. Moreover, a peptide derived from the bacterial elongation factor Tu stimulated S-acylation of its cognate RK, elongation factor-Tu receptor at a paralogous cysteine residue to that mapped in FLS2 (74). All plant RKs possess conserved cysteines homologous to the FLS2/elongation factor-Tu receptor site(s), raising the possibility that many members of this family of kinases undergo dynamic S-acylation. It thus appears very possible that stimulus-dependent S-acylation of protein kinases is more common than might initially be thought.

### Exploiting stimulus-dependent S-acylation for therapeutic benefit

Because stimulus-dependent S-acylation is likely enzymatic, it is also potentially druggable. Accordingly, selectively preventing such modification is now being assessed as a novel therapeutic approach, based on recent exciting findings related to the DLK-JNK3 module. In particular, recent reports suggest that an axonal pool of DLK is S-acylated in response to trophic factor deprivation (49, 75), an event that coincides with increased recruitment of DLK to axonal vesicles (49, 75). DLK’s importance for several forms of neurodegeneration has led to multiple campaigns to develop DLK kinase domain inhibitors (76, 77, 78), but the first such inhibitor to be advanced to a clinical trial caused unintended side effects, including elevated levels of neurofilament proteins in patient plasma, likely reflective of disrupted axonal cytoskeletal integrity (76). Indeed, many patients in this trial reduced their dosage or ceased taking the DLK inhibitor due to symptoms of sensory neuropathy, a condition associated with loss of axonal integrity in DRG sensory neurons (76). Rather than broadly inhibit all cellular pools of DLK, Zhang and co-workers recently used a different approach based on a high content screening assay that assessed nearly 30,000 potential pharmacologic regulators of DLK’s S-acylation–dependent localization (75, 79). They identified two compounds that preferentially inhibit stimulus-dependent changes in DLK’s axonal localization, which is known to be S-acylation–dependent (49, 75). Importantly, these compounds approached the efficacy of a DLK kinase domain inhibitor in preventing prodegenerative retrograde signaling both in cultured neurons and *in vivo* but did not phenocopy the disruption of axonal cytoskeletal integrity seen with global DLK kinase inhibition (75). While these first-generation compounds are unlikely to be sufficiently potent for clinical use, these findings are an exciting proof-of-principle that targeting acute S-acylation events can reduce neurodegeneration and perhaps other forms of disease.

### Writers and erasers—how is S-acylation reversed?

Once S-acylation is demonstrated to occur dynamically and transiently, this raises the question as to how the modification is reversed. The most obvious candidates to mediate de-S-acylation are members of the ABHD family of proteins, several of which have been shown to act as protein de-S-acylases (9, 80, 81). Consistent with this assignation, treatment with the broad spectrum ABHD inhibitor palmostatin B (PalmB) increased the S-acylation of ERK2, although this effect was not phenocopied by more selective ABHD inhibitors, suggesting that multiple de-S-acylases control ERK2 de-S-acylation (73). PalmB also increased S-acylation of Lck in unstimulated cells, although it did not prevent Lck de-S-acylation after Fas stimulation (70). This latter finding suggests that the rapidly S-acylated pool of Lck is de-S-acylated by a PalmB-insensitive de-S-acylase or that another mechanism is at play. For example, if only a small fraction of Lck is S-acylated, it is possible that this S-acylated pool is rapidly degraded without impacting total Lck levels, thus giving the appearance of de-S-acylation. Given that tools to interrogate specific de-S-acylases have emerged only comparatively recently (81, 82), we look forward to additional progress in this area.

### Other cytosolic kinases are S-acylated, with reports of the cellular consequences eagerly awaited

Examples of the functional impact of S-acylation on kinase signaling are thus growing but there are also other S-acylated cytosolic kinases for which an effect on signaling is suggested, but for which the cellular/molecular basis is currently unclear. These include the alpha subunit of adenosine monophosphate-activated protein kinase, whose S-acylation is required for activation of autophagy under glucose starvation conditions (83), and GSK3β, whose S-acylation correlates with decreased phosphorylation at the inhibitory site, Ser-9, and elevated phosphorylation at the activatory site, Tyr-216 (84). However, the direct impact of palmitoyl-site mutation on adenosine monophosphate-activated protein kinase and GSK3β localization and/or activity has yet to be addressed.

There are also other kinases including the mechanistic target of rapamycin kinase, BR serine/threonine kinase, and serine/threonine kinase-16 (85, 86, 87, 88) for which S-acylation has been reported but for which no functional role has yet been ascribed. A recent exhaustive kinase Atlas study also identified several kinases that lack transmembrane or lipid-binding domains, yet which localize to membranes *via* as-yet undescribed mechanisms (89). It is tempting to speculate that S-acylation controls or contributes to such localization. If so, studies of the functional impact of such S-acylation on additional membrane-localized cytosolic kinases are eagerly awaited.

### S-acylation also regulates transmembrane kinases

To this point, we have mainly considered the impact of S-acylation on protein kinases that are initially cytosolic. The S-acylation–dependent increase in hydrophobicity of such kinases and subsequent promotion of their membrane targeting is conceptually straightforward. However, several transmembrane kinases (including the plant RKs described above) are S-acylated and the reason for such S-acylation may be less immediately clear. Although we direct readers to an excellent review on this topic for more information (90), briefly, S-acylation can impact multiple aspects of transmembrane protein localization and function, including conformation of the protein itself and/or targeting to specific microdomains, as well as interaction with other binding partners. Though not investigated extensively, some of these roles of S-acylation have been reported for transmembrane kinases and are functionally important for their signaling. For example, bone morphogenetic protein receptor-1a (BMPR1a), a protein important for brain development, contains three S-acylation sites (91). Two of these sites are essential for BMPR1a function and result in embryonic lethality when mutated. A third site, C180, is not essential for viability but controls BMPR1a mobility in cells and dictates neural stem cell differentiation (91).

**Figure 4:**
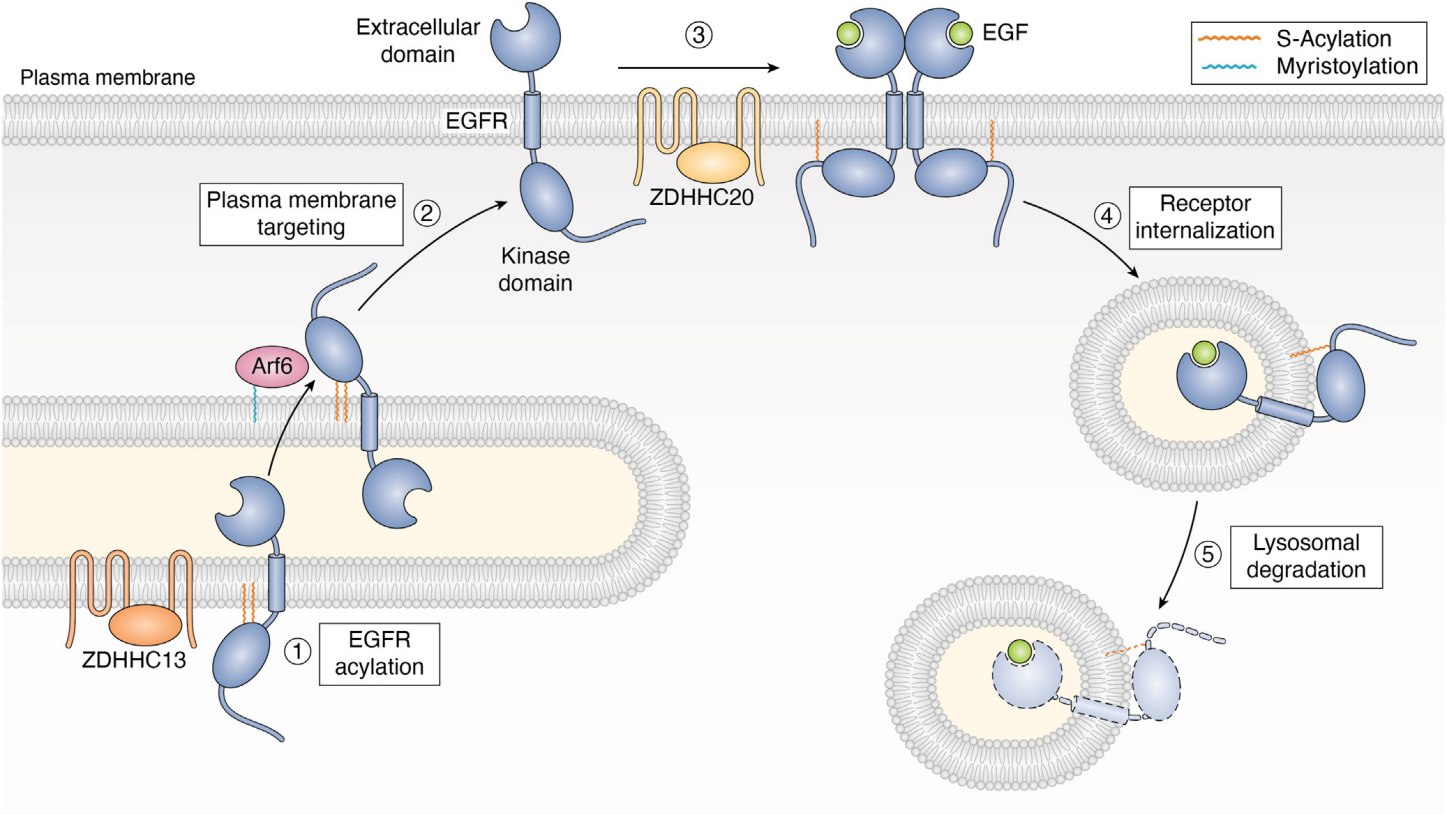
**Roles of S-acylation in EGFR trafficking and signaling.** S-acylation impacts multiple steps of EGFR biology. *Step 1*: EGFR is S-acylated on the Golgi at sites within its kinase domain, likely by ZDHHC13. This initial event promotes EGFR association with the myristoylated small GTPase Arf6. *Step 2*: S-acylation of sites in EGFR’s kinase domain and Arf6 interaction are important for EGFR plasma membrane targeting. These sites may become de-S-acylated once EGFR reaches the plasma membrane. *Step 3*: upon EGF stimulation, EGFR dimerizes and S-acylation of EGFR increases, predominantly at C-terminal sites. This S-acylation event is ascribed to ZDHHC20. Additional phospho-dependent regulation of EGFR and its associated proteins is omitted for clarity. *Steps 4* and *5*: Activated EGFR is internalized and recruited to lysosomes for degradation. However, S-acylation mutant EGFR remains in endosomes (not shown) and can potentially continue to signal. EGFR, epidermal growth factor receptor.

Another example of an S-acylated transmembrane kinase is the epidermal growth factor receptor (EGFR) (92, 93). Several sites within the cytosolic domain of EGFR are reported to be S-acylated. In particular, a cluster of sites within EGFR’s kinase domain that mediate EGFR association with the small GTPase Arf6 are reported to be essential for EGFR trafficking to the plasma membrane. These sites are reported to be S-acylated by ZDHHC13 (93). In contrast, mutation of sites within EGFR’s unstructured C-terminal tail does not affect plasma membrane targeting of EGFR but prolongs EGFR signaling kinetics (94, 95). Loss of ZDHHC20, the PAT reported to S-acylate the EGFR C terminus (92), phenocopies the signaling defect seen in the EGFR S-acylation mutant. Both ZDHHC20 loss of function and the C-terminal S-acylation mutation prevent EGFR trafficking to lysosomes, perhaps allowing EGFR to continue to signal from endosomes (92, 94). This change in regulation is likely important for pathogenic EGFR signaling, because mutating a single C-terminal EGFR S-acylation site reduces tumorigenesis in an adenocarcinoma model (95). Importantly, tumor formation in the same mouse model is similarly reduced by *Zdhhc20* genetic ablation, further implicating ZDHHC20 in EGFR regulation. A model for how S-acylation affects multiple steps of EGFR trafficking and signaling is shown in Figure 4.

Other receptor tyrosine kinases are also S-acylated, including vascular endothelial growth factor receptor-1 and c-Met (96, 97), and S-acylation is required for trafficking of the latter to the plasma membrane. The direct impact of S-acyl site mutation on vascular endothelial growth factor receptor-1 and c-Met signaling has yet to be addressed, but the large number of receptor tyrosine kinases and their key roles in physiological and pathological signaling means that it will be of great interest to determine how widely S-acylation regulates this subgroup of kinases. An intriguing additional possibility, which to our knowledge has not been addressed experimentally, is whether other transmembrane kinase subfamilies (*e.g.*, histidine kinases) found in other evolutionary clades (98) are also subject to S-acylation–dependent regulation.

### Regulation of kinase signaling by S-acylated adaptors

Lastly, S-acylation can regulate localization and/or activity of kinase signaling pathways not only by directly impacting kinases but also by affecting their regulators or binding partners. For example, binding of the S-acylated form of the proprotein convertase subtilisin/kexin type-9 to phosphatase and tensin homolog triggers phosphatase and tensin homolog downregulation and subsequent activation of Akt (99). Similarly, S-acylation of the adaptor A-kinase anchoring protein 79/150 is necessary to localize PKA to neuronal postsynaptic densities and to recycling endosomes (100). Thus, even when a given kinase is not directly S-acylated, its localization and/or activity may still be S-acylation–dependent.

### Interplay of kinase S-acylation with other cellular regulatory mechanisms: Alternative splicing generates specific S-acylated kinase isoforms

S-acylation–dependent regulation may also be coordinated with other intracellular regulatory mechanisms. For example, there are multiple cases in which specific S-acylation sites in kinases and other proteins are generated or removed by alternative splicing. In the case of S-acylation–dependent DLK/JNK signaling, JNK3 is predominantly expressed as a “long” JNK, with a C-terminal extension, but JNK2 is present in both long and short splice forms, of which only the longer isoform is S-acylated (47). This raises several questions, including whether the two JNK2 isoforms are both present in the same cell(s), whether they regulate different substrates and whether the extent of splicing (and hence, potentially the ratio of S-acylated:non-S-acylated JNK2) differs in different cell types and/or during development.

Alternative splicing also generates specifically S-acylated isoforms of other kinases. One isoform of Bruton’s tyrosine kinase (BTK) has an extended N terminus containing two S-acylation sites. Mutation of these sites in this BTK-C isoform reduces BTK-C tyrosine phosphorylation, raising the possibility that S-acylation of BTK-C impacts the known role of this kinase, for example, in apoptosis resistance in epithelial tumors (101). However, more work will be required to test this hypothesis. In addition, apoptosis-associated tyrosine kinase 1, also known as lemur tail kinase-1 (LMTK1), has an S-acylated LMTK1A isoform that localizes to endosomes in an S-acylation–dependent manner (102). It is an intriguing possibility that LMTK1A’s role in axonal outgrowth (103), which potentially involves LMTK1A action at or close to the axonal membrane, also requires it’s S-acylation, although this has yet to be addressed.

### A bonus feature of S-acylation–dependent kinase regulation; minimized phosphorylation of inappropriate substrates

The striking effects of S-acylation on kinase localization and signaling might be thought to be sufficient to account for the importance of this regulatory mechanism. However, S-acylation can also potentially shape or control signaling by a given kinase or pathway in multiple additional ways, any or all of which may impact the outcome(s) of an initial signal. For example, several studies now suggest that one role of S-acylation is not only to localize kinases close to their physiological substrate(s) but also to prevent phosphorylation of other, perhaps inappropriate, target proteins. We previously suggested that the coupling of DLK’s vesicular localization and kinase activity might explain findings that DLK loss predominantly impacts pathological but not physiological JNK signaling (46, 52). This may be because de-S-acylated DLK is not only absent from vesicles but is also enzymatically inactive and is thus likely incapable of phosphorylating inappropriate cytosolic substrates (46). Likewise, S-acylation keeps LIMK1 close to its upstream, membrane-localized activators Cdc42/PAK3 (23). LIMK1’s key substrate, the actin filament-severing protein cofilin, would thus be predicted to be phosphorylated and inhibited only in this juxtamembrane region while remaining active closer to the center of a cell (23). This mechanism would be predicted to favor the juxtamembrane actin filament polymerization needed for directional cell growth, while ensuring that filaments do not hyperextend or polymerize in inappropriate locations.

Conversely, there are examples of the potential danger to a cell if a kinase retains activity despite loss of S-acylation. For example, de-S-acylated SFKs can drive chromosome mis-segregation when targeted to the nucleus (104), creating a situation in which loss of physiological SFK membrane localization not only blunts physiological signaling but also drives pathological signaling. Likewise, while S-acylation of CK1γ3 is critical for its ability to mediate Wnt/β−catenin signaling (35), deacylated CK1γ3 is cytosolic and hyperactive, and phosphorylates and inactivates the ceramide transport binding protein (36).

Lastly, though not directly demonstrated, uncoupling of S-acylation–dependent kinase localization and activity may have direct pathological consequences. For example, the *JAK1* oncogenic mutation, V658F, overrides the S-acylation dependence of JAK1 activity, raising the possibility that JAK1-V658F is active in cellular locations beyond those to which WT JAK1 is restricted (69). It is tempting to speculate that such unrestricted signaling contributes to this mutant’s oncogenic properties (105, 106, 107).

### Emergent properties of S-acylation–dependent kinase localization: Temporal shaping and salience of S-acyl-kinase–dependent vesicular signals

**Figure 5:**
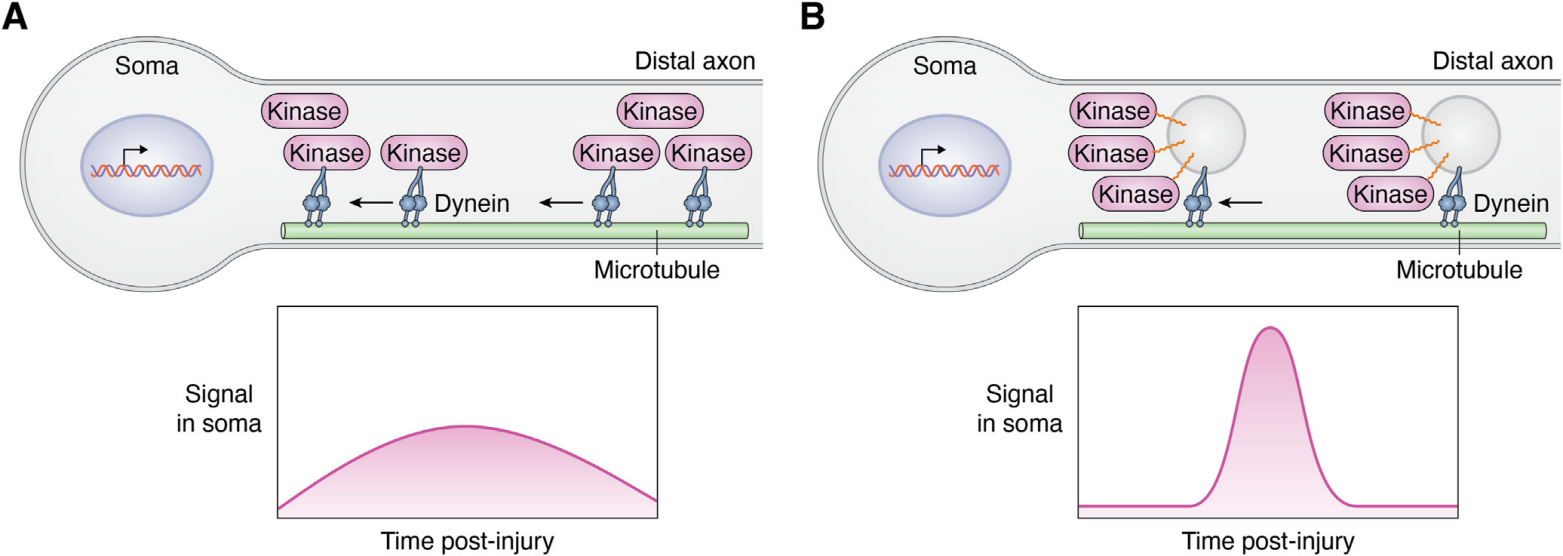
**Potential regulation of salience and/or kinetics of retrograde axonal signals by S-acylated retrograde signaling kinase(s).***A*, individual molecules of an otherwise soluble kinase are trafficked retrogradely from axons toward the neuronal soma. Arrival of individual kinases in the neuronal soma is asynchronous. *Lower graph*: potential signal in soma (*e.g.*, transcription factor activation) driven by asynchronous kinase arrival. *B*, in contrast, a retrograde signaling vesicle likely carries many molecules of an S-acylated kinase. Arrival of a retrograde signaling vesicle in the soma will simultaneously deliver many molecules of kinase. *Lower panel*: potential signal in soma driven by synchronous kinase arrival.

**Figure 6:**
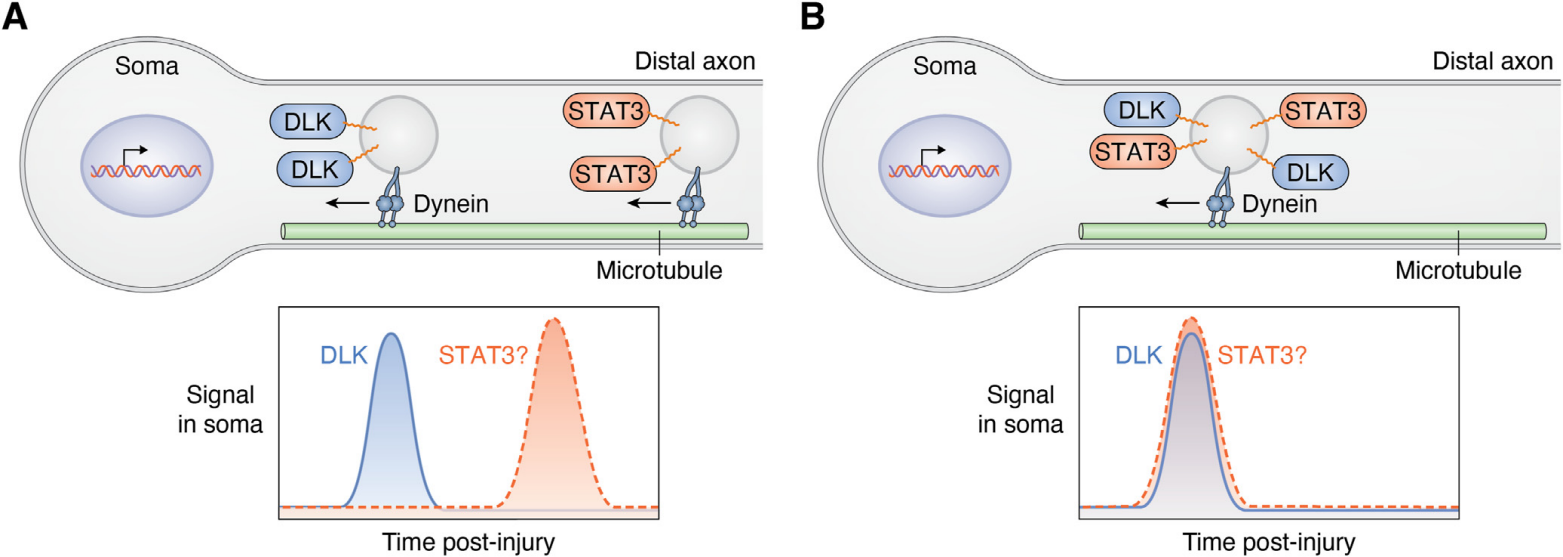
**Potential integration of information from multiple retrograde signaling pathways by S-acylation.***A*, the protein kinase DLK and the transcription factor STAT3 are both important retrograde signaling proteins and both are S-acylated. In scenario 1, retrograde signals mediated by S-acylated DLK and STAT3 are carried on separate populations of axonal vesicles. *Lower panel*: asynchronous arrival of DLK-positive and STAT3-positive vesicles in the neuronal soma would trigger asynchronous activation of downstream events (*e.g.*, transcriptional responses) driven by the two pathways. *B*, scenario 2: the finding that DLK is necessary for STAT3 retrograde axonal transport (110) raises the possibility that DLK and STAT3 cotraffic on the same vesicles. *Lower panel*: arrival of a DLK-/STAT3-double-positive vesicle in the neuronal soma may synchronously activate downstream events driven by the two pathways. DLK, dual leucine-zipper kinase.

Other effects of S-acylation on kinase signaling are perhaps less immediately apparent and might even be described as “emergent properties” of this modification. For example, live imaging studies of vesicle-based hitchhiking described above suggest that not only DLK and JNK3 but also MKK4 and JIP3 all traffic together on individual single vesicles (46, 47) (Fig. 2*B*). Based on the imaging set up used, each vesicle visualized in these studies likely carries numerous molecules of each kinase, a conclusion consistent with other studies assessing copy numbers of vesicular proteins (108, 109). By cotrafficking together, multiple copies of a retrogradely transported DLK-MKK4-JNK3 module would therefore reach the soma simultaneously as a “bolus,” or signalosome (Fig. 5). While difficult to assess experimentally, it would appear possible that the consolidated arrival of multiple kinase molecules on such a signalosome would drive a more substantial and/or temporally distinct transcriptional response profile, compared with a series of sporadically arriving individual kinase molecules (Fig. 5, *A* and *B*). Moreover, a given vesicle could potentially carry not only DLK-JNK3–dependent signals but also information from other signaling pathways. For example, the transcription factor STAT3 is also important for retrograde injury signaling and is known to traffic retrogradely in a DLK-dependent manner in axons (110, 111), perhaps also due to S-acylation (112). It is thus possible that DLK/JNK and STAT3-dependent retrograde signals, and potentially elements of other signaling pathways, are conveyed on a “master” signalosome vesicle that is assembled *via* S-acylation (and/or additional mechanisms) of its individual components. Such a signalosome could potentially integrate information from multiple pathways, driving a unique signature response upon arriving in the cell soma (Fig. 6, *A* and *B*).

Finally, S-acylation–dependent tethering of multiple kinases may also ensure the fidelity of a given signal over long times and/or distances. For example, MAPK pathway enzymes are readily inactivated by dephosphorylation, and dephosphorylation of a retrogradely transported DLK-JNK3 signalosome prior to its arrival at the cell soma would mean it might as well not have made the long journey back along the axon at all. However, an additional property of the DLK-JNK3 module is its ability to establish a positive feedback loop, whereby active JNK3 phosphorylates and further activates DLK itself (47, 113). This feedback phosphorylation maintains the DLK-JNK3 module in a highly active state that would be predicted to be less susceptible to phosphatase-mediated inactivation (Fig. 2*B*). Interestingly, this feedback loop is not seen with the related, non-S-acylated, p38 MAPK, or with related, non-S-acylated mitogen-activated protein kinase kinase kinases and may be an additional reason why the S-acylated DLK-JNK3 module has emerged as the key pathway to convey retrograde long-distance signals in mammalian axons (46, 52, 55). Together, these emergent mechanisms may facilitate the ability of neurons, and perhaps other cell types, to mount robust and coordinated responses to diverse extracellular signals.

### S-acylation–dependent regulation of other signaling proteins and pathways

We have focused this review on the regulation of protein kinases, but S-acylation also impacts many other families of intracellular signaling enzymes. A full documentation of these roles is beyond the scope of this overview, even more so because additional findings in this realm are constantly being reported. However, we briefly mention some key families of S-acylated signaling proteins and summarize recently defined roles of this modification related to other aspects of intracellular signaling.

Perhaps most relevant to our focus on protein kinases is the question of whether protein phosphatases are also S-acylated. Indeed, specific isoforms of protein phosphatase 1 (PP1) were proteomically identified as being fatty acylated, a finding confirmed in follow-up targeted experiments (114). Although the type of fatty acyl attachment was not initially identified, the same subunits were identified in multiple other palmitoyl-proteomic studies, suggesting that PP1 subunits are indeed S-acylated. However, the functional impact of S-acylation on PP1 targeting and signaling has yet to be addressed. More recently, a less well-studied isoform of the phosphatase calcineurin subunit Aβ1 (CNAβ1) was also reported to be S-acylated, in contrast to the canonical CNAβ2 subunit (115). S-acylation of CNAβ1 allows calcineurin to interact with a distinct set of membrane-bound proteins, including phosphatidylinositol 4-kinase (PI4K). Moreover, calcineurin inhibitors impair PI4K signaling that is normally induced by activation of Gq-coupled GPCRs (115). It is thus tempting to speculate that CNAβ1 S-acylation normally facilitates calcineurin-dependent regulation of PI4K. These findings suggest that S-acylation–dependent effects on localization, signaling hub assembly, and downstream functional roles are seen with other families of signaling enzymes and are not limited to protein kinases.

Reports of S-acylation–dependent phosphatase regulation are nonetheless sparse, compared with kinases, although we note several possible explanations for this disparity. Most importantly, S-acylation of several other phosphatase subunits (of both serine/threonine and tyrosine phosphatase subfamilies), and an array of their regulatory subunits and other interactors, is annotated in proteomic databases, but few of these reports have been followed up in targeted studies (116). It is thus possible that lipidation of these interactors, by S-acylation or perhaps another lipid modification, facilitates dephosphorylation of S-acylated kinases by the catalytic phosphatase subunit. Protein–protein interactions of phosphatase subunits with nonlipidated but membrane-associated binding partners could also play a role. Lastly, regulation may be achieved *via* de-S-acylation of the kinase and then dephosphorylation by a cytosolic phosphatase(s). Given that these different possibilities are not mutually exclusive; the potential reversibility of S-acylated kinase signaling by phosphatases is a fertile area for additional investigation.

Key components of other signaling pathways are also known to be regulated *via* S-acylation and this is particularly true for GPCR-dependent signaling. Many subfamilies of GPCRs are S-acylated, including receptors for melanocortin, chemokines, serotonin, epinephrine (α1, α2, and β2 subtypes of adrenergic receptor) and several other neurotransmitters. In addition, G protein alpha subunits themselves were amongst the first S-acylated signaling proteins to be identified (117, 118). For GPCRs, S-acylation is often necessary for both correct localization and functional signaling (119). Likewise, mutation of G alpha subunit S-acylation sites affects both targeting and signaling ability (120). However, for both these families of protein, it appears that the effect on signaling can potentially be explained by the effect on localization. When compared to kinases, effects of S-acylation on GPCRs and G alpha subunits described to this point are thus more akin to the regulation of SFKs and LIMK1 and do not extend to alterations in intrinsic signaling ability, as reported for DLK, GPCR kinase 6, and JAK1. We direct readers to excellent recent reviews on this topic for further information (119, 121).

More recently, key steps in numerous other signaling pathways have also been reported to be S-acylation–dependent. Two such examples are the nucleotide oligomerization domain–like receptors 1 and 2 (nucleotide oligomerization domain 1/2), which activate immune signaling pathways after sensing bacterial peptidoglycans (122) and stimulator of interferon response cGAMP interactor 1, which is important for interferon response activation triggered by exposure to DNA pathogens (123). Again, more detailed reviews provide additional information on these pathways (124, 125). It will be of great interest to determine the extent to which other S-acylated proteins that activate or influence intracellular signaling, ranging from metabolic enzymes to viral pathogens, for example (126, 127, 128), exert their effects in an S-acylation–dependent manner. An equally important and interesting issue will be to determine whether the impact of S-acylation on these other proteins and pathways can be explained by effects on the same properties of localization, signalosome assembly, and/or intrinsic activity described for kinases or reflects previously undescribed roles for this modification.

### Current knowledge, challenges, and future opportunities

There are now numerous examples of S-acylation events that are critically important for intracellular signaling. Surprisingly, most events categorized to this point do not appear to take advantage of the potential dynamic regulation of S-acylation, but multiple examples of such regulation have recently been reported, and may be more readily observed given the increasing number of tools to monitor and control dynamic S-acylation and de-S-acylation (for example 82, 129, 130). In addition, as conditional KO mouse lines lacking specific PATs are generated and characterized (54, 122, 131), the ability to define roles of S-acylation in specific tissues or cell types is also increasing. Likewise, S-acylation–deficient knock in lines are also now emerging (132, 133), providing new insights into the functional roles of individual S-acylation events *in vivo*.

There are still challenges, however. Pharmacologic tools to probe the importance of S-acylation are somewhat limited, with the most widely used compound 2-bromopalmitate suffering from poor selectivity and toxicity issues (130, 134). Despite recent development of improved inhibitors of S-acylation (130), the ability to pharmacologically target specific ZDHHC-PATs and even specific S-acylation events would greatly benefit the field. The situation with de-S-acylase inhibitors is more promising, with several selective compounds reported (82, 135, 136). Nonetheless, the best studied de-S-acylase inhibitor PalmB, while effective, is somewhat labile in cell culture and its hydrophobicity may affect its delivery *in vivo* (137). Developing chemical genetic approaches to drive or inhibit specific S-acylation and/or de-S-acylation events could help circumvent these issues.

In addition, progress in identifying S-acylation–dependent roles of intracellular signaling enzymes has largely relied on a combination of large-scale proteomic studies and painstaking case-by-case follow-up or targeted studies. Advances in nonradioactive S-acylation assays (138, 139, 140) and site-mapping methods (92, 141, 142) have facilitated these efforts, but it would be extremely beneficial if medium-throughput approaches could be developed to more broadly identify S-acylation–dependent signaling events and/or their functional roles. For a field that has always been closely associated with technological advances, it is thus particularly exciting that “bump-and-hole” methods, first developed to define the substrates of protein kinases (143) have recently been expanded to, and optimized for, PATs (144, 145). Thus far, these studies have focused on using “bumped” lipids to identify substrates for transfected “hole-mutant” PATs in a gain-of-function manner. However, it will be of great interest to know whether “bumped” PAT inhibitors might be developed and combined with knocked-in “hole-mutant” PATs to begin to identify substrates (including, but not limited to, protein kinases and other signaling enzymes) and roles for endogenous PATs, as was done successfully for kinases themselves *e.g.*, (146). The possible use of CRISPR-based technology to develop “hole-mutant” cell lines, perhaps even in patient-derived inducible pluripotent cells to model disease conditions, is another exciting future direction.

Its clear relevance both to fundamental biology across the evolutionary spectrum and to a broad array of pathological conditions provides strong motivation to more fully define the control of intracellular signaling by S-acylation. With a suite of exciting new methods and reagents being developed, the next few years of progress in this field are eagerly anticipated.

## Conflicts of interest

A Patent Cooperation Treaty (PCT) Application related to a screening method described in this review was jointly filed by Temple University and Shriners Hospitals for Children. Author G. M. T. (inventor) is named in the PCT. The PCT filing and submission of this manuscript are being overseen by Temple University in accordance with its appropriate policies. Author X. Z. declares that she has no conflicts of interest with the contents of this article.
